# Crystal structure, Hirshfeld surface analysis and physicochemical characterization of bis­[4-(di­methyl­amino)­pyridinium] di-μ-chlorido-bis[di­chlorido­mercurate(II)]

**DOI:** 10.1107/S2056989019013124

**Published:** 2019-10-03

**Authors:** Fatma Garci, Hela Ferjani, Hammouda Chebbi, Mariem Ben Jomaa, Mohamed Faouzi Zid

**Affiliations:** a University of Tunis El Manar, Faculty of Sciences of Tunis, Laboratory of Materials, Crystal Chemistry and Applied Thermodynamics, 2092 El Manar II, Tunis, Tunisia; b Chemistry Department, College of Science, IMSIU (Imam Mohammad Ibn Saud Islamic University), Riyadh 11623, Kingdom of Saudi Arabia

**Keywords:** crystal structure, chloro­mercurate(II) salt, 4-di­methyl­amino­pyridium, Hirshfeld surface, fingerprint plots, hybrid compound

## Abstract

The title mol­ecular salt, (C_7_H_11_N_2_)_2_[Hg_2_Cl_6_], crystallizes with two 4-(di­methyl­amino)­pyridinium cations (*A* and *B*) and two half hexa­chlorido­dimercurate(II) anions in the asymmetric unit. The organic cations exhibit essentially the same features with an almost planar pyridyl ring (r.m.s. deviations of 0.0028 and 0.0109 Å), which forms an inclined dihedral angle with the dimethyamino group [3.06 (1) and 1.61 (1)°, respectively]. The di­methyl­amino groups in the two cations are planar, and the C—N bond lengths are shorter than that in 4-(di­methyl­amino)­pyridine. In the crystal, mixed cation–anion layers lying parallel to the (010) plane are formed through N—H⋯Cl hydrogen bonds and adjacent layers are linked by C—H⋯Cl hydrogen bonds, forming a three-dimensional network. The analyses of the calculated Hirshfeld surfaces confirm the relevance of the above inter­molecular inter­actions, but also serve to further differentiate the weaker inter­molecular inter­actions formed by the organic cations and inorganic anions, such as π–π and Cl⋯Cl inter­actions. The powder XRD data confirms the phase purity of the crystalline sample. Furthermore, the vibrational absorption bands were identified by IR spectroscopy and the optical properties were studied by using optical UV–visible absorption spectroscopy.

## Abstract 2

The two independent organic cations in the asymmetric unit of the chloro­mercurate(II) salt exhibit essentially the same features with almost planar pyridinium and di­methyl­amino groups. In the crystal, N—H⋯Cl and C—H⋯Cl hydrogen bonds as well as *π–π* and Cl⋯Cl inter­actions link the organic cations and inorganic anions into a three-dimensional network.

## Chemical context   

Hybrid organic–inorganic materials have been widely studied in recent years for their promising applications in different fields, including catalysis, magnetism and optics and for their luminescence properties (Clément *et al.*, 1994 ▸; Rabu *et al.*, 2001 ▸; Hu *et al.*, 2003 ▸; Morris *et al.*, 2008 ▸). However, owing to the confinement of the inorganic layers, the organic cations have to possess the right ionic bond and steric hindrance, as well as hydrogen bonds, to fit the coordination environment provided by the inorganic framework for stabilization of these organic–inorganic hybrid systems.

Hybrids based on mercury have been synthesized and characterized with simple, different techniques, thanks to their self-assembling character (Mitzi *et al.*, 2001 ▸) and are very inter­esting both for fundamental physics exploration such as electronic confinement (Wei *et al.*, 2015 ▸) or as low-dimensional magnetic systems (Fersi *et al.*, 2015 ▸) and diversify the field of technological applications.

A number of chloro­mercurate(II) complexes have been shown to exhibit ferroelectric behaviour (Mitsui & Nakamura, 1990 ▸) and inter­est has focused on the mechanism of the ferroelectric–paraelectric phase transition (White, 1963 ▸; Körfer *et al.*, 1988 ▸; Jiang *et al.*, 1995 ▸; Liesegang *et al.*, 1995 ▸) for which structural information is crucial. In addition, the ability of the anions in this class of compounds to exhibit a wide range of geometry, stoichiometry and connectivity has long been known (Grdenic, 1965 ▸). This flexibility is a result of the large volume and spherical charge distribution of the Hg^2+^ ion, which are a consequence of the filled 4*f* and 5*d* electron shells. Moreover, organic–inorganic materials with pyridine and its derivatives as template agents have led to the preparation of some materials with inter­esting physical properties (Aakeröy *et al.*, 2000 ▸; Prince *et al.*, 2003 ▸) and biological activities (Bossert *et al.*, 1981 ▸; Wang *et al.*, 1989 ▸).
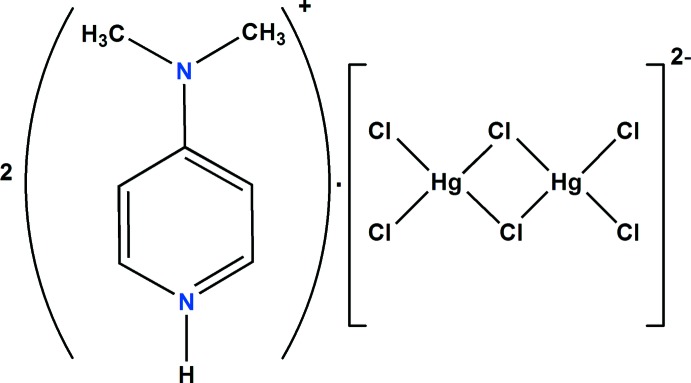


As part of our continuing investigation of new hybrid compounds containing an organic cation and an inorganic anion such as CrO_4_^2−^ (Chebbi *et al.*, 2000 ▸; Chebbi & Driss, 2001 ▸, 2002*a* ▸,*b* ▸, 2004 ▸), Cr_2_O_7_^2−^ (Chebbi *et al.*, 2016 ▸, Ben Smail *et al.*, 2017 ▸), NO_3_^−^ (Chebbi *et al.*, 2014 ▸, 2018 ▸) and ClO_4_^−^ (Chebbi *et al.*, 2017 ▸; Ben Jomaa *et al.*, 2018 ▸), we report in this work the crystal structure, the Hirshfeld surface analysis and the physicochemical characterization of a new organic chloro­mercurate(II), (C_7_H_11_N_2_)_2_[Hg_2_Cl_6_] (I).

## Structural commentary   

**Figure 1:**
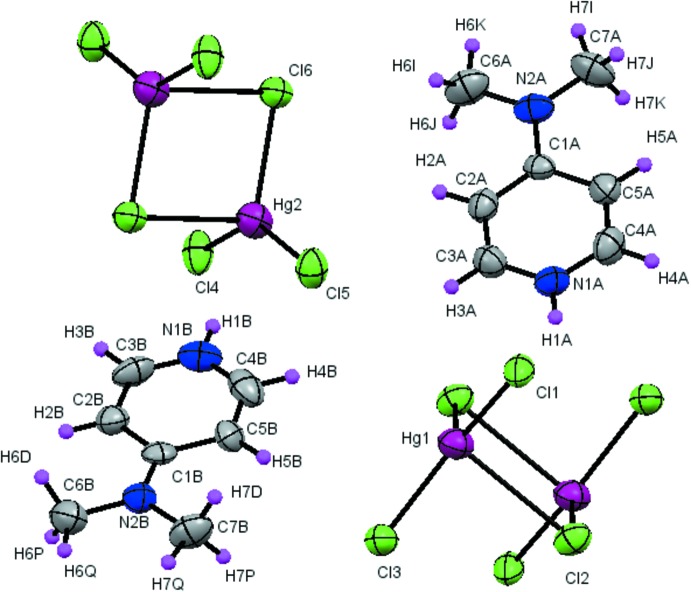
The mol­ecular structure of (I). Atomic displacement parameters for the non-H atoms are drawn at the 50% probability level. Unlabelled atoms are related to labelled ones by the symmetry operation −*x* + 1, −*y*, −*z* + 2.

The asymmetric unit of the title compound comprises two 4-(di­methyl­amino)­pyridinium cations (*A* and *B*), and two half [Hg_2_Cl_6_]^2−^ anions (Fig. 1 ▸). The two independent [Hg_2_(1,2)Cl_6_]^2−^ anions are found to adopt a centrosymmetric arrangement with terminal Cl1—Hg1—Cl3 and Cl4—Hg2—Cl5 angles of 141.4 (1)° and 141.7 (1)° respectively. Each anion appears to be a distorted edge-shared bi­tetra­hedron, similar to that reported by Larock *et al.* (1987 ▸), with its center of mass coincident with a crystallographic center of symmetry. The two independent Hg⋯Cl bridging distances are 2.539 (2) and 2.542 (2) Å, leading to a slightly asymmetric bridging system as has been found in most structures containing the [Hg_2_Cl_6_]^2−^ moiety (Linden *et al.*, 1999 ▸; Zabel *et al.*, 2008 ▸). In each anion, the two terminal Hg—Cl bonds are quite short [Hg1—Cl1 = 2.371 (2) and Hg1—Cl3 = 2.380 (2) Å, Hg2—Cl4 = 2.367 (3) and Hg2—Cl5 = 2.392 (2) Å] with a Cl1—Hg1—Cl2 and Cl4—Hg2—Cl6 angles of 112.01 (9) and 112.72 (10)°, respectively. Assessment of the organic geometrical features shows that they exhibit essentially the same features with an almost planar pyridyl ring (r.m.s. deviation = 0.0028 and 0.0109 Å for C1*A*–C5*A*/N1*A* and C1*B*–C5*B*/N1*B*, respectively), which forms an inclined dihedral angle with the dimethyamino group [3.06 (1) and 1.61 (1)°, respectively]. The di­methyl­amino groups in the two cations are planar and the C—N bond lengths [1.357 (11) Å for *A* and 1.326 (11) Å for *B*] are shorter than that in 4-di­methyl­amino­pyridine [1.367 (2) Å; Ohms & Guth, 1984 ▸]. These findings indicate the presence of strong conjugation between the di­methyl­amino group and the pyridine ring. The C3*A*—N1*A*—C4*A* [121.2 (8)°] and C3*B*—N1*B*—C4*B* [119.8 (9)°] bond angles are wider than that in pyridine (116.94°; Sørensen *et al.*, 1974 ▸), which indicates that the pyridine ring N atom is protonated. Examination of the C—C(N) distances and C—C—C (N), C—N—C angles in the 4-(di­methyl­amino)­pyridinium dications (*A* and *B*) shows no significant difference from those obtained in other organic materials associated with the same organic groups (Chao *et al.*, 1977 ▸; Mustaqim *et al.*, 2005 ▸).

**Figure 2:**
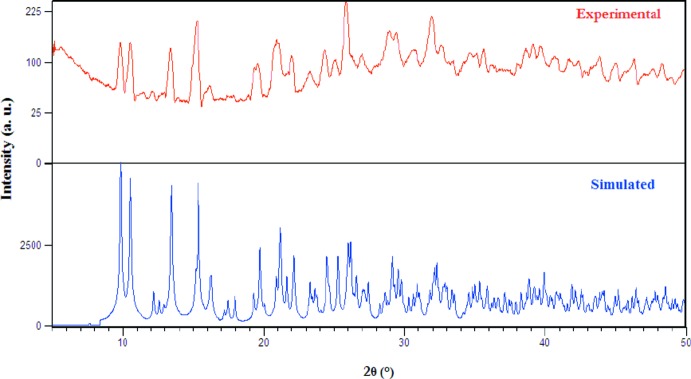
Experimental and simulated powder XRD patterns of (I).

The experimental powder X-ray diffraction pattern of the title compound, (C_7_H_11_N_2_)_2_[Hg_2_Cl_6_] is in good agreement with that simulated (Fig. 2 ▸). This indicates the purity of the synthesized product and confirms the crystal data used.

## Supra­molecular features   

**Figure 3:**
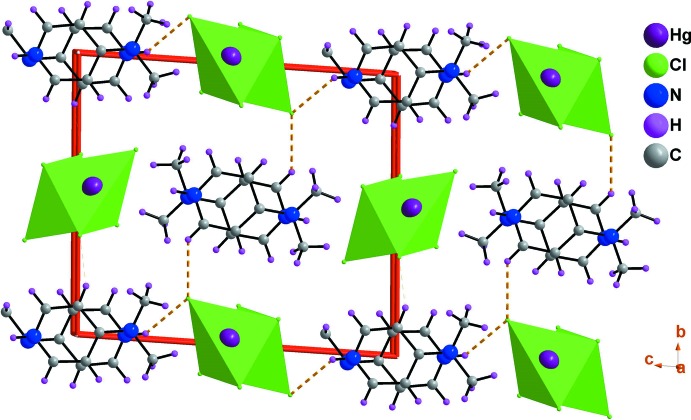
Structure of (I) viewed along the *a* axis showing the succession of mixed layers parallel to the (010) plane. The orange dotted lines indicate hydrogen bonds.

**Figure 4:**
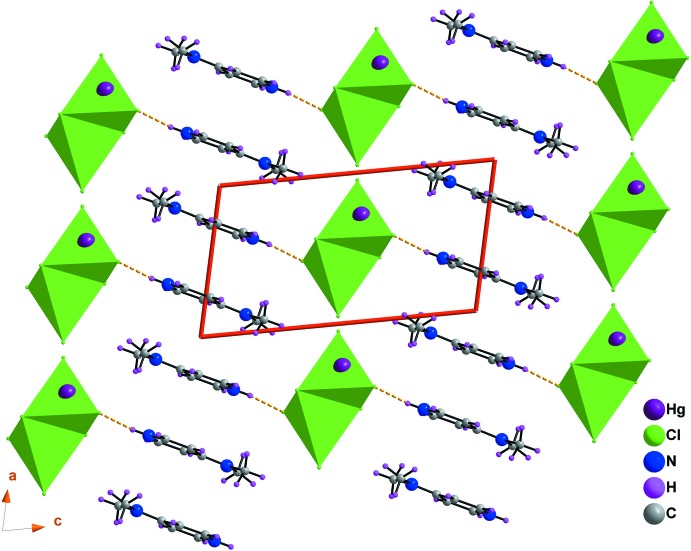
A view of the supra­molecular mixed layer in the *ac* plane of (I), showing the alternating organic and inorganic columns parallel to the [100] direction. The orange dotted lines indicate N—H⋯Cl hydrogen bonds.

**Figure 5:**
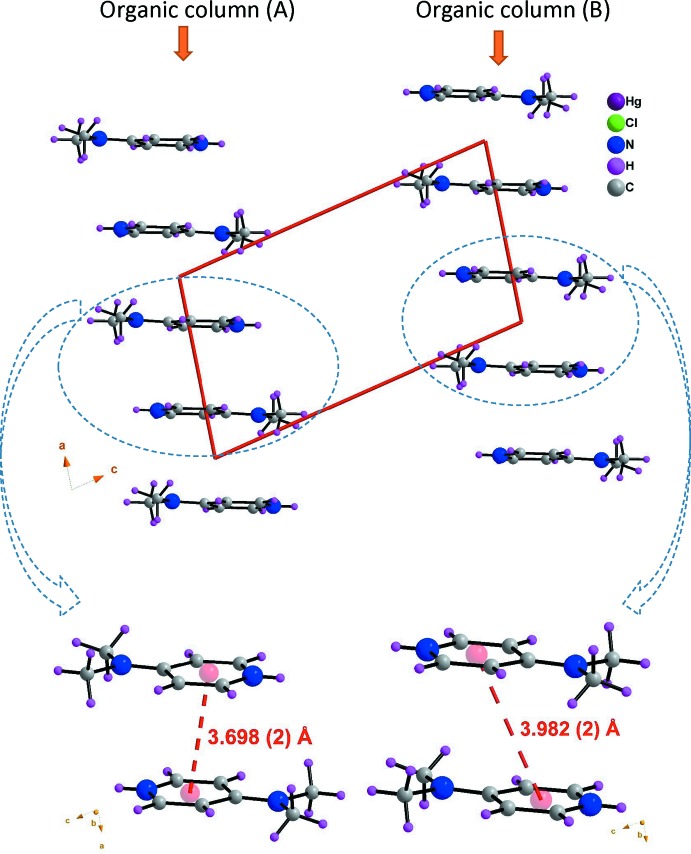
π–π stacking inter­actions between the nearest aromatic organic cation neighbors into two types of organic columns (*A* or *B*).

**Figure 6:**
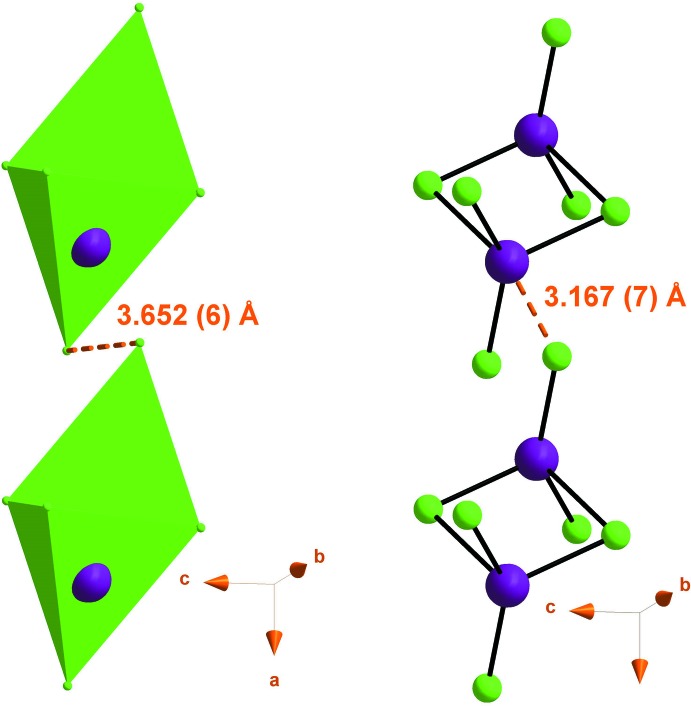
Cl⋯Cl and Hg⋯Cl inter­actions between hexa­chlorido­dimercurate(II) anions dispersed parallel to the *a* axis in the inorganic column.

In the crystal structure, mixed cation–anion layers lying parallel to the (010) plane are formed through N—H⋯Cl hydrogen bonds and adjacent layers are linked by C—H⋯Cl hydrogen bonds, forming a three-dimensional network (Table 1 ▸, Fig. 3 ▸). A mixed layer is formed by alternating of organic and inorganic columns parallel to the [100] direction (Fig. 4 ▸). The cations (*A* or *B*) inter­act *via* offset face-to-face π–π stacking inter­actions, leading to two types of organic columns formed by the cations (*A* or *B*) with centroid-centroid distances of 3.698 (2) and 3.982 (2) Å, respectively (Fig. 5 ▸) (Janiak, 2000 ▸; Ben Moussa *et al.*, 2018 ▸). Similarly, the hexa­chlorido­dimercurate(II) anions are dispersed parallel to the *a* axis whose cohesion is ensured by Cl⋯Cl [3.652 (6) Å] and Hg⋯Cl [3.167 (7) Å] weak inter­actions (Sumanesh *et al.*, 2016 ▸; Ben Moussa *et al.*, 2019*a* ▸,*b* ▸; Fig. 6 ▸).

## Vibrational study   

**Figure 7:**
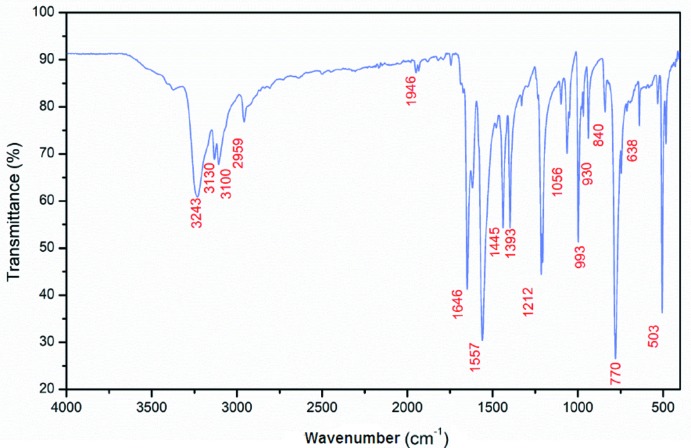
Infrared spectrum of (I).

The obtained FT–IR spectrum for the studied hexa­chlorido­dimercurate(II) salt is depicted in Fig. 7 ▸. Detailed assignment of all bands observed in the infrared spectrum of the 4-(di­methyl­amino)­pyridinium cation in the title compound is based on the comparison with other compounds associated to the same cation (Koleva *et al.*, 2008 ▸; Hu *et al.*, 2012 ▸). In the region of high frequencies, the bands at 3243, 3130, 3100, 2959 cm^−1^ are due to the stretching vibrations of the N—H and C—H bonds. The band at 1646 cm^−1^ is assigned to the N—H bending mode. The bands at 1557 and 1445 cm^−1^ are attributed to the C=C and C=N stretching modes of the pyridine ring. The absorption band located at 1212 cm^−1^ corresponds to the ν(C—N) and ν(C—C) modes. The band at 1056 cm^−1^ can be attributed to the δ(C—C) mode. The remaining bands in the range 1000 to 500 cm^−1^ are assigned to γ(C—C), γ(C—H) and γ(C—N) out-of-plane bending modes.

## Optical properties and frontier mol­ecular orbitals   

**Figure 8:**
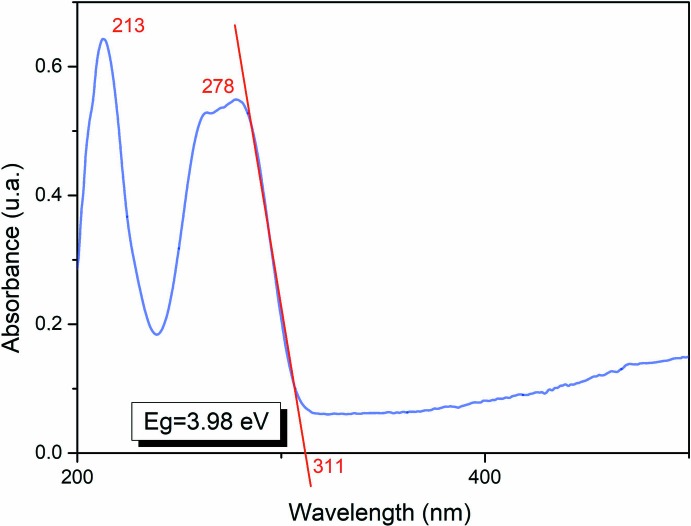
UV–vis spectrum of (I). The inset shows the experimental energy band gap obtained from the absorption edge wavelength.

**Figure 9:**
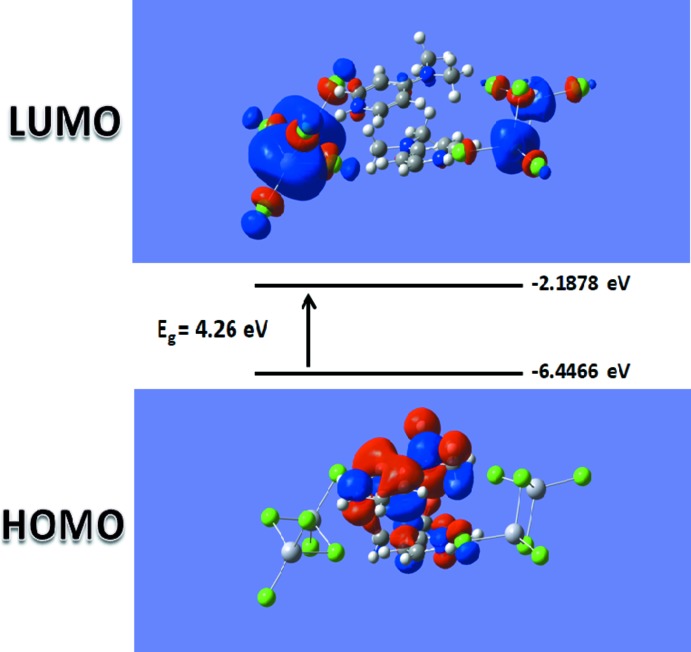
HOMO–LUMO mol­ecular orbitals showing the ground to excited state electronic transitions for (I).

Optical absorption (OA) measurement of the title compound was performed at ambient temperature in an ethanol solution (10^−4^ *M*). As shown in Fig. 8 ▸, the OA spectrum exhibits two distinct absorption bands around 213 and 278 nm assigned to the π→π* absorption bands of the 4-(di­methyl­amino)­pyridinium cations. Thus, the experimental band-gap energy obtained from the absorption edge wavelength is about 3.98 eV. This band-gap value indicates that the grown crystal exhibits semiconductor behavior (Rosencher & Vinter, 2002 ▸). The highest occupied mol­ecular orbital (HOMO) and the lowest unoccupied mol­ecular orbital (LUMO), known as frontier orbitals, obtained with a B3LYP/6-311G+(d,p) [H, C, N, Cl]–LANL2DZ [Hg] level calculation are illustrated in Fig. 9 ▸. The HOMO is mainly delocalized at the pyridine ring system. After excitation, the charge is localized on the hexa­chlorido­dimercurate(II) moieties, as depicted in the LUMO. The calculated HOMO–LUMO energy gap (4.26 eV) is shifted from the experimental value, which may be attributed to solvent effects, compared to the gas-phase calculation.

## Hirshfeld surface analysis   

**Figure 10:**
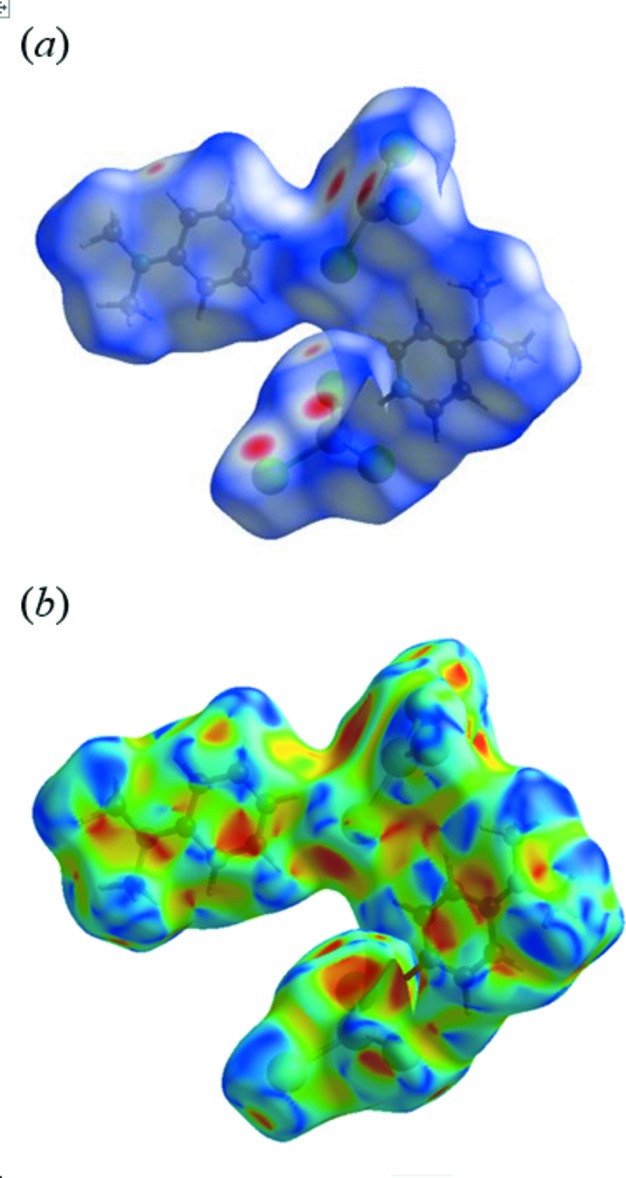
View of the Hirshfeld surfaces for (I) mapped over (*a*) *d*_norm_ and (*b*) shape-index, displaying the inter­molecular inter­actions.

A Hirshfeld surface analysis (Spackman & Jayatilaka, 2009 ▸) and the associated two-dimensional fingerprint plots (McKinnon *et al.*, 2007 ▸) were performed with *CrystalExplorer17* (Turner *et al.*, 2017 ▸) to investigate the inter­molecular inter­actions in the title compound. Fig. 10 ▸ *a* illustrates the Hirshfeld surface mapped over *d*_norm_, which was plotted with a colour scale of −0.211 to 1.132 a.u. with a standard (high) surface resolution. The red spots highlight the inter­atomic contacts including the N—H⋯Cl and C—H⋯Cl hydrogen bonds.

The shape-index of the Hirshfeld surface is a tool to visualize the *π–π* stacking by the presence of adjacent red and blue triangles; if there are no adjacent red and/or blue triangles, then there are no *π–π* inter­actions. Fig.10*b* clearly suggests that π–π inter­actions are present in the title hexa­chlorido­dimercurate(II) salt.

**Figure 11:**
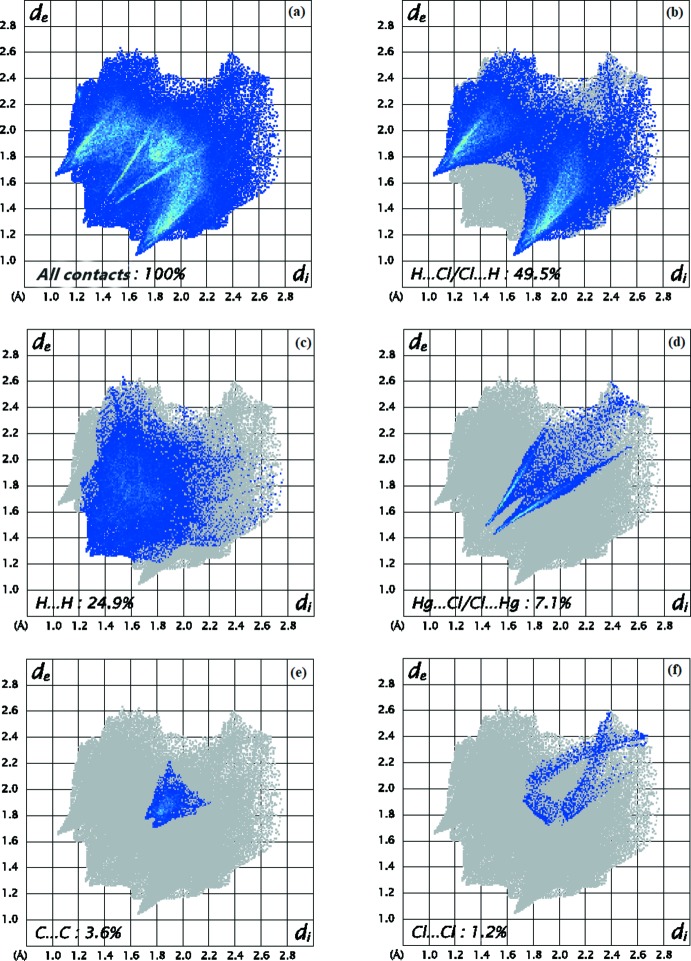
Full two-dimensional fingerprint plots for (I), showing (*a*) all inter­actions, and delineated into (*b*) H⋯Cl/Cl⋯H, (*c*) H⋯H, (*d*) Hg⋯Cl/Cl⋯Hg, (*e*) C⋯C and (*f*) Cl⋯Cl inter­actions. The *d*_i_ and *d*_e_ values are the closest inter­nal and external distances (in Å) from a given point on the Hirshfeld surface.

**Figure 12:**
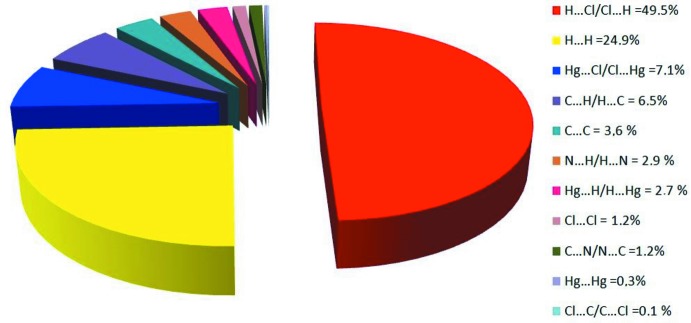
Relative contribution (%) of various inter­molecular inter­actions to the Hirshfeld surface area.

Fig. 11 ▸ *a* shows the two-dimensional fingerprint of all contacts contributing to the Hirshfeld surface. In Fig. 11 ▸ *b*, with two symmetrical wings on the left and right sides illustrate the H⋯Cl/Cl⋯H inter­actions with a contribution of 49.5%. Fig. 11 ▸ *c* illustrates the two-dimensional fingerprint plot of (*d*_i_, *d*_e_) points related to H⋯H contacts, which represent a 24.9% contribution. Furthermore, there are Hg⋯Cl/Cl⋯Hg (7.1%; Fig. 11 ▸ *d*), C⋯C (3.6%; Fig. 11 ▸ *e*) and Cl⋯Cl (1.2%; Fig. 11 ▸ *f*) contacts. Fig. 12 ▸ shows the percentage contributions of the various contacts in the title structure.

## Synthesis and crystallization   

The title compound was synthesized by dissolving 2 mmol (241 mg) of 4-di­methyl­amino­pyridine 98% (Sigma–Aldrich) in an HCl 36–38% (Sigma–Aldrich) aqueous solution and 1 mmol (273 mg) of mercury(II) chloride HgCl_2_ (Merck) in ethanol in a molar ratio of 2:1. The mixture was then stirred for 2 h. The resulting aqueous solution was filtered and then evaporated at room temperature, which finally led to the growth of parallelepipedic colourless crystals after one day.

## Refinement   

Crystal data, data collection and structure refinement details are summarized in Table 2 ▸. H atoms were placed in calculated positions, with N—H = 0.86 Å and C—H = 0.93 or 0.96 Å. *U*_iso_(H) values were constrained to be 1.5*U*_eq_ of the carrier atom for methyl H atoms, and 1.2*U*_eq_ for the remaining H atoms. The (111) and (121) reflections were omitted owing to bad disagreement.

## Supplementary Material

Crystal structure: contains datablock(s) I. DOI: 10.1107/S2056989019013124/vm2222sup1.cif

Structure factors: contains datablock(s) I. DOI: 10.1107/S2056989019013124/vm2222Isup2.hkl

CCDC reference: 1911692

Additional supporting information:  crystallographic information; 3D view; checkCIF report

## Figures and Tables

**Table 1:** Hydrogen-bond geometry (Å, °)

*D*—H⋯*A*	*D*—H	H⋯*A*	*D*⋯*A*	*D*—H⋯*A*
N1*A*—H1*A*⋯Cl3	0.86	2.54	3.239 (8)	140
N1*B*—H1*B*⋯Cl5	0.86	2.46	3.195 (10)	145
C2*B*—H2*B*⋯Cl1^i^	0.93	2.82	3.634 (11)	147
C3*A*—H3*A*⋯Cl5	0.93	2.75	3.485 (11)	136

Symmetry code: (i) 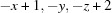.

**Table 2:** Experimental details

Crystal data	
Chemical formula	(C_7_H_11_N_2_)_2_[Hg_2_Cl_6_]
*M*_r_	860.23
Crystal system, space group	Triclinic, *P* 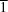
Temperature (K)	293
*a*, *b*, *c* (Å)	7.6558 (3), 11.8961 (5), 13.5853 (4)
α, β, γ (°)	82.950 (3), 76.072 (3), 76.339 (4)
*V* (Å^3^)	1164.07 (8)
*Z*	2
Radiation type	Mo *K*α
μ (mm^−1^)	13.87
Crystal size (mm)	0.72 × 0.24 × 0.18

Data collection	
Diffractometer	Enraf–Nonius CAD-4
Absorption correction	ψ scan (North *et al.*, 1968 ▸)
*T*_min_, *T*_max_	0.53, 0.99
No. of measured, independent and observed [*I* > 2σ(*I*)] reflections	7139, 5875, 3913
*R*_int_	0.040
(sin θ/λ)_max_ (Å^−1^)	0.671

Refinement	
*R*[*F* ^2^ > 2σ(*F* ^2^)], *wR*(*F* ^2^), *S*	0.055, 0.153, 1.03
No. of reflections	5875
No. of parameters	236
H-atom treatment	H-atom parameters constrained
Δρ_max_, Δρ_min_ (e Å^−3^)	3.41, −3.00

Computer programs: *CAD-4 EXPRESS* (Duisenberg, 1992 ▸; Macíček & Yordanov, 1992 ▸), *XCAD4* (Harms & Wocadlo, 1995 ▸), *SHELXT2018* (Sheldrick, 2015*a* ▸), *SHELXL2018* (Sheldrick, 2015*b* ▸), *DIAMOND* (Brandenburg, 2006 ▸), *Mercury* (Macrae *et al.*, 2006 ▸), *WinGX* (Farrugia, 2012 ▸) and *publCIF* (Westrip, 2010 ▸).

## supplementary crystallographic information

### Crystal data

**Table:**

(C_7_H_11_N_2_)_2_[Hg_2_Cl_6_]	*Z* = 2
*M**_r_* = 860.23	*F*(000) = 792
Triclinic, *P*1	*D*_x_ = 2.454 Mg m^−^^3^
*a* = 7.6558 (3) Å	Mo *K*α radiation, λ = 0.71073 Å
*b* = 11.8961 (5) Å	Cell parameters from 25 reflections
*c* = 13.5853 (4) Å	θ = 10–15°
α = 82.950 (3)°	µ = 13.87 mm^−^^1^
β = 76.072 (3)°	*T* = 293 K
γ = 76.339 (4)°	Parallelepiped, colorless
*V* = 1164.07 (8) Å^3^	0.72 × 0.24 × 0.18 mm

### Data collection

**Table:**

Enraf–Nonius CAD-4 diffractometer	3913 reflections with *I* > 2σ(*I*)
Radiation source: fine-focus sealed tube	*R*_int_ = 0.040
Graphite monochromator	θ_max_ = 28.5°, θ_min_ = 2.3°
ω/2θ scans	*h* = −10→2
Absorption correction: ψ scan (North *et al.*, 1968)	*k* = −15→15
*T*_min_ = 0.53, *T*_max_ = 0.99	*l* = −18→18
7139 measured reflections	2 standard reflections every 120 reflections
5875 independent reflections	intensity decay: 1%

### Refinement

**Table:**

Refinement on *F*^2^	Hydrogen site location: inferred from neighbouring sites
Least-squares matrix: full	H-atom parameters constrained
*R*[*F*^2^ > 2σ(*F*^2^)] = 0.055	*w* = 1/[σ^2^(*F*_o_^2^) + (0.0933*P*)^2^] where *P* = (*F*_o_^2^ + 2*F*_c_^2^)/3
*wR*(*F*^2^) = 0.153	(Δ/σ)_max_ = 0.001
*S* = 1.03	Δρ_max_ = 3.41 e Å^−^^3^
5875 reflections	Δρ_min_ = −3.00 e Å^−^^3^
236 parameters	Extinction correction: SHELXL2018 (Sheldrick, 2015b), Fc^*^=kFc[1+0.001xFc^2^λ^3^/sin(2θ)]^-1/4^
0 restraints	Extinction coefficient: 0.0058 (5)

### Special details

**Table:**

Geometry. All esds (except the esd in the dihedral angle between two l.s. planes) are estimated using the full covariance matrix. The cell esds are taken into account individually in the estimation of esds in distances, angles and torsion angles; correlations between esds in cell parameters are only used when they are defined by crystal symmetry. An approximate (isotropic) treatment of cell esds is used for estimating esds involving l.s. planes.

### Fractional atomic coordinates and isotropic or equivalent isotropic displacement parameters (Å^2^)

**Table:**

	*x*	*y*	*z*	*U*_iso_*/*U*_eq_	
Hg1	0.73846 (6)	0.52983 (3)	0.96401 (3)	0.04867 (15)	
Hg2	0.70787 (6)	0.02376 (3)	0.53548 (3)	0.05017 (16)	
Cl3	0.7012 (4)	0.6752 (2)	0.82926 (18)	0.0507 (6)	
Cl5	0.5562 (4)	0.1596 (2)	0.66140 (18)	0.0518 (6)	
Cl2	0.4827 (3)	0.5982 (2)	1.11547 (17)	0.0489 (6)	
Cl6	0.5814 (4)	0.1067 (2)	0.37887 (17)	0.0487 (5)	
Cl1	0.9450 (4)	0.3586 (2)	1.0069 (2)	0.0507 (6)	
Cl4	0.9497 (4)	−0.1420 (2)	0.5038 (2)	0.0544 (6)	
N1B	0.3722 (13)	0.0129 (9)	0.8551 (6)	0.055 (2)	
H1B	0.423341	0.022921	0.791911	0.067*	
N1A	0.6907 (12)	0.5280 (8)	0.6462 (6)	0.047 (2)	
H1A	0.665775	0.537818	0.710142	0.056*	
N2B	0.1208 (12)	−0.0324 (7)	1.1557 (6)	0.0441 (18)	
C1B	0.2047 (12)	−0.0182 (8)	1.0590 (6)	0.0359 (19)	
C5B	0.2580 (14)	0.0880 (8)	1.0172 (8)	0.043 (2)	
H5B	0.237846	0.149257	1.057848	0.051*	
N2A	0.8004 (12)	0.4827 (8)	0.3413 (6)	0.047 (2)	
C2A	0.7198 (13)	0.4105 (8)	0.5161 (8)	0.042 (2)	
H2A	0.712530	0.340021	0.496158	0.050*	
C4B	0.3388 (16)	0.0977 (10)	0.9166 (9)	0.058 (3)	
H4B	0.372259	0.167244	0.889581	0.069*	
C1A	0.7683 (12)	0.4958 (8)	0.4424 (6)	0.0352 (18)	
C2B	0.2482 (14)	−0.1070 (9)	0.9916 (7)	0.043 (2)	
H2B	0.223027	−0.179339	1.015900	0.052*	
C3B	0.3261 (15)	−0.0884 (10)	0.8918 (8)	0.052 (3)	
H3B	0.347796	−0.147079	0.848196	0.063*	
C5A	0.7751 (14)	0.6032 (9)	0.4768 (8)	0.047 (2)	
H5A	0.805230	0.663582	0.430389	0.056*	
C4A	0.7372 (15)	0.6156 (9)	0.5779 (8)	0.050 (2)	
H4A	0.742924	0.684838	0.600944	0.061*	
C3A	0.6831 (14)	0.4271 (9)	0.6157 (7)	0.045 (2)	
H3A	0.652280	0.368100	0.663649	0.054*	
C7B	0.0765 (17)	0.0644 (11)	1.2239 (8)	0.061 (3)	
H7Q	0.016344	0.039805	1.290807	0.091*	
H7P	0.188302	0.085789	1.227041	0.091*	
H7D	−0.003486	0.129990	1.197662	0.091*	
C6B	0.0727 (16)	−0.1412 (10)	1.1987 (8)	0.058 (3)	
H6Q	0.013266	−0.134628	1.269144	0.087*	
H6D	−0.009493	−0.159869	1.162861	0.087*	
H6P	0.182338	−0.201411	1.192574	0.087*	
C7A	0.8505 (17)	0.5720 (11)	0.2661 (8)	0.063 (3)	
H7I	0.867226	0.546304	0.199482	0.094*	
H7J	0.754609	0.640812	0.274933	0.094*	
H7K	0.963334	0.588643	0.273377	0.094*	
C6A	0.8007 (17)	0.3711 (11)	0.3057 (8)	0.065 (3)	
H6K	0.825473	0.376560	0.232724	0.097*	
H6I	0.894286	0.311834	0.328549	0.097*	
H6J	0.682564	0.351849	0.332384	0.097*	

### Atomic displacement parameters (Å^2^)

**Table:**

	*U*^11^	*U*^22^	*U*^33^	*U*^12^	*U*^13^	*U*^23^
Hg1	0.0531 (3)	0.0467 (2)	0.0452 (2)	−0.00220 (18)	−0.01806 (17)	−0.00119 (16)
Hg2	0.0507 (3)	0.0470 (3)	0.0479 (2)	−0.00196 (18)	−0.00822 (17)	−0.00665 (17)
Cl3	0.0702 (17)	0.0420 (12)	0.0451 (12)	−0.0111 (11)	−0.0240 (11)	−0.0015 (10)
Cl5	0.0602 (15)	0.0440 (13)	0.0483 (12)	−0.0159 (11)	0.0017 (11)	−0.0101 (10)
Cl2	0.0486 (13)	0.0604 (15)	0.0393 (11)	−0.0136 (11)	−0.0069 (10)	−0.0111 (10)
Cl6	0.0551 (14)	0.0543 (14)	0.0414 (11)	−0.0190 (11)	−0.0166 (10)	0.0044 (10)
Cl1	0.0547 (14)	0.0363 (11)	0.0709 (16)	−0.0105 (10)	−0.0348 (12)	0.0018 (11)
Cl4	0.0431 (13)	0.0373 (12)	0.0800 (18)	−0.0075 (10)	−0.0079 (12)	−0.0065 (11)
N1B	0.052 (5)	0.072 (7)	0.038 (4)	−0.005 (5)	−0.012 (4)	−0.002 (4)
N1A	0.057 (5)	0.053 (5)	0.034 (4)	−0.016 (4)	−0.011 (4)	−0.006 (4)
N2B	0.049 (5)	0.048 (5)	0.035 (4)	−0.012 (4)	−0.009 (3)	−0.004 (3)
C1B	0.030 (4)	0.043 (5)	0.035 (4)	0.002 (3)	−0.018 (3)	−0.002 (4)
C5B	0.049 (5)	0.028 (4)	0.050 (5)	−0.001 (4)	−0.018 (4)	0.000 (4)
N2A	0.041 (4)	0.067 (6)	0.033 (4)	−0.011 (4)	−0.007 (3)	−0.005 (4)
C2A	0.040 (5)	0.035 (5)	0.053 (5)	−0.006 (4)	−0.015 (4)	−0.007 (4)
C4B	0.050 (6)	0.047 (6)	0.072 (7)	−0.005 (5)	−0.021 (5)	0.017 (5)
C1A	0.031 (4)	0.042 (5)	0.034 (4)	−0.007 (4)	−0.013 (3)	0.001 (4)
C2B	0.046 (5)	0.044 (5)	0.043 (5)	−0.006 (4)	−0.019 (4)	−0.004 (4)
C3B	0.052 (6)	0.061 (7)	0.046 (5)	0.004 (5)	−0.019 (5)	−0.026 (5)
C5A	0.049 (6)	0.043 (5)	0.051 (6)	−0.013 (4)	−0.018 (5)	0.003 (4)
C4A	0.053 (6)	0.038 (5)	0.061 (6)	−0.004 (4)	−0.013 (5)	−0.014 (5)
C3A	0.046 (5)	0.048 (6)	0.041 (5)	−0.013 (4)	−0.008 (4)	0.004 (4)
C7B	0.061 (7)	0.080 (8)	0.042 (5)	−0.014 (6)	−0.008 (5)	−0.019 (5)
C6B	0.064 (7)	0.063 (7)	0.051 (6)	−0.019 (6)	−0.019 (5)	0.005 (5)
C7A	0.066 (7)	0.074 (8)	0.041 (5)	−0.013 (6)	−0.006 (5)	0.013 (5)
C6A	0.068 (8)	0.081 (9)	0.048 (6)	−0.014 (7)	−0.011 (5)	−0.020 (6)

### Geometric parameters (Å, º)

**Table:**

Hg1—Cl1	2.371 (2)	C2A—C3A	1.344 (14)
Hg1—Cl3	2.380 (2)	C2A—C1A	1.386 (13)
Hg1—Cl2	2.539 (2)	C2A—H2A	0.9300
Hg1—Cl2^i^	2.975 (2)	C4B—H4B	0.9300
Hg2—Cl4	2.367 (3)	C1A—C5A	1.429 (13)
Hg2—Cl5	2.392 (2)	C2B—C3B	1.357 (15)
Hg2—Cl6	2.542 (2)	C2B—H2B	0.9300
Hg2—Cl6^ii^	2.934 (3)	C3B—H3B	0.9300
N1B—C4B	1.329 (15)	C5A—C4A	1.352 (15)
N1B—C3B	1.340 (15)	C5A—H5A	0.9300
N1B—H1B	0.8600	C4A—H4A	0.9300
N1A—C3A	1.334 (13)	C3A—H3A	0.9300
N1A—C4A	1.362 (14)	C7B—H7Q	0.9600
N1A—H1A	0.8600	C7B—H7P	0.9600
N2B—C1B	1.326 (12)	C7B—H7D	0.9600
N2B—C6B	1.445 (14)	C6B—H6Q	0.9600
N2B—C7B	1.492 (13)	C6B—H6D	0.9600
C1B—C2B	1.410 (13)	C6B—H6P	0.9600
C1B—C5B	1.429 (14)	C7A—H7I	0.9600
C5B—C4B	1.360 (15)	C7A—H7J	0.9600
C5B—H5B	0.9300	C7A—H7K	0.9600
N2A—C1A	1.357 (11)	C6A—H6K	0.9600
N2A—C7A	1.435 (14)	C6A—H6I	0.9600
N2A—C6A	1.468 (15)	C6A—H6J	0.9600
			
Cl1—Hg1—Cl3	141.44 (10)	C2A—C1A—C5A	117.1 (8)
Cl1—Hg1—Cl2	112.01 (9)	C3B—C2B—C1B	121.0 (10)
Cl3—Hg1—Cl2	106.16 (9)	C3B—C2B—H2B	119.5
Cl1—Hg1—Cl2^i^	93.63 (8)	C1B—C2B—H2B	119.5
Cl3—Hg1—Cl2^i^	88.63 (8)	N1B—C3B—C2B	121.1 (9)
Cl2—Hg1—Cl2^i^	94.45 (7)	N1B—C3B—H3B	119.4
Cl4—Hg2—Cl5	141.70 (10)	C2B—C3B—H3B	119.4
Cl4—Hg2—Cl6	112.72 (10)	C4A—C5A—C1A	119.1 (10)
Cl5—Hg2—Cl6	105.06 (9)	C4A—C5A—H5A	120.5
Cl4—Hg2—Cl6^ii^	95.28 (8)	C1A—C5A—H5A	120.5
Cl5—Hg2—Cl6^ii^	87.53 (8)	C5A—C4A—N1A	120.7 (9)
Cl6—Hg2—Cl6^ii^	94.73 (7)	C5A—C4A—H4A	119.6
Hg1—Cl2—Hg1^i^	85.55 (7)	N1A—C4A—H4A	119.6
Hg2—Cl6—Hg2^ii^	85.27 (7)	N1A—C3A—C2A	120.3 (9)
C4B—N1B—C3B	119.8 (9)	N1A—C3A—H3A	119.9
C4B—N1B—H1B	120.1	C2A—C3A—H3A	119.9
C3B—N1B—H1B	120.1	N2B—C7B—H7Q	109.5
C3A—N1A—C4A	121.2 (8)	N2B—C7B—H7P	109.5
C3A—N1A—H1A	119.4	H7Q—C7B—H7P	109.5
C4A—N1A—H1A	119.4	N2B—C7B—H7D	109.5
C1B—N2B—C6B	121.7 (9)	H7Q—C7B—H7D	109.5
C1B—N2B—C7B	120.2 (9)	H7P—C7B—H7D	109.5
C6B—N2B—C7B	118.1 (8)	N2B—C6B—H6Q	109.5
N2B—C1B—C2B	122.2 (9)	N2B—C6B—H6D	109.5
N2B—C1B—C5B	121.8 (9)	H6Q—C6B—H6D	109.5
C2B—C1B—C5B	116.1 (9)	N2B—C6B—H6P	109.5
C4B—C5B—C1B	118.6 (9)	H6Q—C6B—H6P	109.5
C4B—C5B—H5B	120.7	H6D—C6B—H6P	109.5
C1B—C5B—H5B	120.7	N2A—C7A—H7I	109.5
C1A—N2A—C7A	122.1 (9)	N2A—C7A—H7J	109.5
C1A—N2A—C6A	120.1 (9)	H7I—C7A—H7J	109.5
C7A—N2A—C6A	117.6 (9)	N2A—C7A—H7K	109.5
C3A—C2A—C1A	121.6 (9)	H7I—C7A—H7K	109.5
C3A—C2A—H2A	119.2	H7J—C7A—H7K	109.5
C1A—C2A—H2A	119.2	N2A—C6A—H6K	109.5
N1B—C4B—C5B	123.3 (11)	N2A—C6A—H6I	109.5
N1B—C4B—H4B	118.4	H6K—C6A—H6I	109.5
C5B—C4B—H4B	118.4	N2A—C6A—H6J	109.5
N2A—C1A—C2A	122.8 (9)	H6K—C6A—H6J	109.5
N2A—C1A—C5A	120.0 (9)	H6I—C6A—H6J	109.5

Symmetry codes: (i) −*x*+1, −*y*+1, −*z*+2; (ii) −*x*+1, −*y*, −*z*+1.

### Hydrogen-bond geometry (Å, º)

**Table:**

*D*—H···*A*	*D*—H	H···*A*	*D*···*A*	*D*—H···*A*
N1*A*—H1*A*···Cl3	0.86	2.54	3.239 (8)	140
N1*B*—H1*B*···Cl5	0.86	2.46	3.195 (10)	145
C2*B*—H2*B*···Cl1^iii^	0.93	2.82	3.634 (11)	147
C3*A*—H3*A*···Cl5	0.93	2.75	3.485 (11)	136

Symmetry code: (iii) −*x*+1, −*y*, −*z*+2.
